# Positional cranial deformation and domain-specific neurodevelopment in small-for-gestational-age infants: a cross-sectional study

**DOI:** 10.3389/fpubh.2026.1936897

**Published:** 2026-10-27

**Authors:** Jiang Zhu, Dan Wu, Lingyan Chen, Daqian Zhu, Yangyi Chen, Masaya Tachibana, Xia Hong, Yoshiyuki Hirano, Jinjin Chen

**Affiliations:** 1 Child Health Care Medical Division, Shanghai Children’s Hospital, School of Medicine, Shanghai Jiao Tong University, Shanghai, China; 2 Department of Cognitive Neuroscience, Donders Center for Brain, Cognition and Behaviour, Radboud University Medical Center, Nijmegen, Netherlands; 3 Department of Child Development, United Graduate School of Child Development, The University of Osaka, Suita, Japan; 4 Molecular Research Center for Children's Mental Development, United Graduate School of Child Development, The University of Osaka, Suita, Japan; 5 Department of Pediatrics, The University of Osaka Graduate School of Medicine, Suita, Japan; 6 Research Center for Child Mental Development, Chiba University, Chiba, Japan; 7 United Graduate School of Child Development, The University of Osaka, Kanazawa University, Hamamatsu University School of Medicine, Chiba University, and University of Fukui, Suita, Osaka, Japan; 8 Center for Cognitive Behavioral Therapy, Chiba University Hospital, Chiba, Japan; 9 NHC Key Laboratory of Medical Embryogenesis and Developmental Molecular Biology & Shanghai Key Laboratory of Embryo and Reproduction Engineering, Shanghai, China; 10 College of Health Science and Technology, Shanghai Jiao Tong University School of Medicine, Shanghai, China

**Keywords:** developmental surveillance, infant, neurodevelopment, positional cranial deformation, small for gestational age

## Abstract

**Background:**

Infants born small for gestational age (SGA) face a higher likelihood of divergent neurodevelopmental trajectories. Positional cranial deformation is an early and observable clinical feature that has been associated with developmental differences in general populations, but its neurodevelopmental relevance in SGA infants remains unclear. This study investigated the associations between positional cranial deformation and domain-specific neurodevelopmental performance among SGA infants.

**Methods:**

This cross-sectional analysis was conducted within a clinical follow-up cohort of SGA infants. A total of 156 infants who completed both three-dimensional cranial morphology assessment and Gesell Developmental Schedules (GDS) evaluation were included; the upper eligibility limit was 18 months of age. Assessments were concentrated in early infancy, with median ages of 6.36 months (IQR, 4.80–9.18) at cranial scanning and 6.90 months (IQR, 6.12–10.08) at GDS assessment. Multiple linear regression models examined the associations between cranial shape and developmental quotient (DQ) scores.

**Results:**

Overall cranial deformation was associated with lower developmental quotient scores in the fine motor and adaptive behavior domains after false discovery rate correction (*P*_FDR_ < 0.05). Exploratory subtype analyses suggested similar patterns for brachycephaly and plagiocephaly, particularly in fine motor and adaptive behavior domains. The direction and magnitude of associations were generally consistent in sensitivity analyses.

**Conclusion:**

Cranial deformation in SGA infants was associated with differences in developmental performance in fine motor and adaptive behavior domains. Exploratory subtype analyses suggested nominal associations for brachycephaly and plagiocephaly, but these associations were no longer significant after multiple-comparison correction. These findings suggest that cranial morphology may provide an accessible clinical indicator for developmental monitoring during SGA follow-up.

## Introduction

Positional cranial deformation (PCD) represents one of the most prevalent skull abnormalities in infancy and early childhood, including plagiocephaly, brachycephaly, and scaphocephaly (1, 2). After the “Back to Sleep” campaign became widely implemented, PCD has been reported more frequently, with some cases persisting into adolescence (3–5).

PCD is often initially perceived as a cosmetic issue, with craniofacial asymmetry or occipital flattening as its most apparent manifestations. Such cranial changes often raise considerable concern among caregivers and may be related to poorer quality of life for infants and their families (6). In severe cases, PCD may co-occur with musculoskeletal dysfunction (e.g., torticollis), visual abnormalities (e.g., strabismus), or otorhinolaryngological issues (7, 8). Beyond physical symptoms, an increasing amount of evidence suggests that PCD may also be associated with differences in early neurodevelopmental performance. Infants younger than 4 months with PCD have lower Test of Infant Motor Performance (TIMP) scores than those with normal cranial shapes, and lower TIMP scores appear to correlate with greater severity of deformation (2). Furthermore, children of school age who previously had moderate-to-severe PCD have been reported to show poorer academic performance and persistently lower cognitive and motor scores (9, 10). Collectively, these observational findings suggest that PCD may be an early and readily observable clinical feature associated with developmental vulnerability, although they do not establish PCD as a cause of subsequent developmental difficulties.

Small for gestational age (SGA) is generally defined as birth weight below the 10th percentile for gestational age and sex. SGA is a size-at-birth classification that includes both constitutionally small infants and infants whose small size may reflect impaired fetal growth. Although outcomes vary considerably within this heterogeneous population, infants born SGA are, as a group, at increased risk of less favorable postnatal growth and neurodevelopmental outcomes (11–13). Direct evidence that SGA status independently increases the occurrence of PCD remains limited. Nevertheless, lower birth weight has been associated with positional plagiocephaly in healthy term infants (3), and head positional preference and the attainment of fewer early motor milestones have also been associated with PCD (14). Given that SGA infants may have vulnerabilities in early growth and motor development that overlap with factors associated with PCD, PCD may be particularly relevant in this population.

Against this background, we conceptualized PCD primarily as a clinical feature of developmental vulnerability within the SGA population, rather than as an established independent risk factor or a direct consequence of SGA-related factors. We therefore examined whether PCD was associated with concurrent domain-specific developmental performance among infants in an SGA follow-up cohort. We paired three-dimensional cranial imaging with standardized developmental assessments to examine the associations of overall PCD and specific cranial deformation subtypes with developmental performance across different domains. Few studies have examined these associations specifically within an SGA population. The findings may help clarify whether the observation of PCD during SGA follow-up should prompt closer attention to the infant’s developmental status.

## Methods

### Study design and participants

Data for this analysis were drawn from an SGA infant follow-up cohort maintained at Shanghai Children’s Hospital. The cohort comprised infants with pre-existing longitudinal follow-up data and infants subsequently enrolled and followed prospectively through routine growth and developmental surveillance. This cross-sectional analysis included infants assessed between September 2021 and April 2026 who had undergone both cranial morphology assessment and Gesell developmental evaluation by 18 months of age, with no more than 6 months between the two assessments. The 18-month threshold represented the upper eligibility limit rather than the typical assessment age of the analytic cohort. Because both assessments were performed as part of routine clinical care, their exact timing and order varied according to clinical scheduling. Therefore, the present analysis focused on associations between cranial morphology and developmental performance, rather than temporal or causal relationships. Baseline demographic and clinical variables were obtained from routine follow-up records and parental questionnaires, including infant sex, gestational age at birth, birth weight, maternal education level, major neonatal conditions used for exclusion, and pregnancy complications.

Inclusion criteria were as follows: (1) birth weight below the 10th percentile for gestational age and sex according to the INTERGROWTH-21st international standards (15); (2) parents who provided relevant information and signed informed consent for participation; (3) availability of three-dimensional (3D) cranial scan data; and (4) completion of the Gesell Developmental Schedules.

Exclusion criteria were as follows: (1) major genetic disorders, congenital conditions, or neonatal disorders known to affect neurodevelopment (e.g., chromosomal abnormalities, confirmed metabolic diseases, neonatal asphyxia or hypoxic–ischemic encephalopathy, intracranial hemorrhage, neonatal respiratory distress syndrome, or other similar conditions); (2) craniosynostosis (confirmed by physical examination or imaging assessment); (3) severe sensory impairments (e.g., bilateral hearing loss or visual impairment); (4) recent acute illness, including pneumonia, dysentery, fever persisting for more than 7 days during the 2-week period before enrollment, or diarrhea occurring more than 5 times daily for more than 5 days.

Figure 1 illustrates the participant selection process. The source cohort included 1,027 SGA infants who underwent cranial scanning during the study period. Of these, 216 completed the Gesell assessment. Among them, 156 also had complete 3D cranial morphology data and met all other eligibility criteria, and were retained for the final analysis.

**Figure 1:**
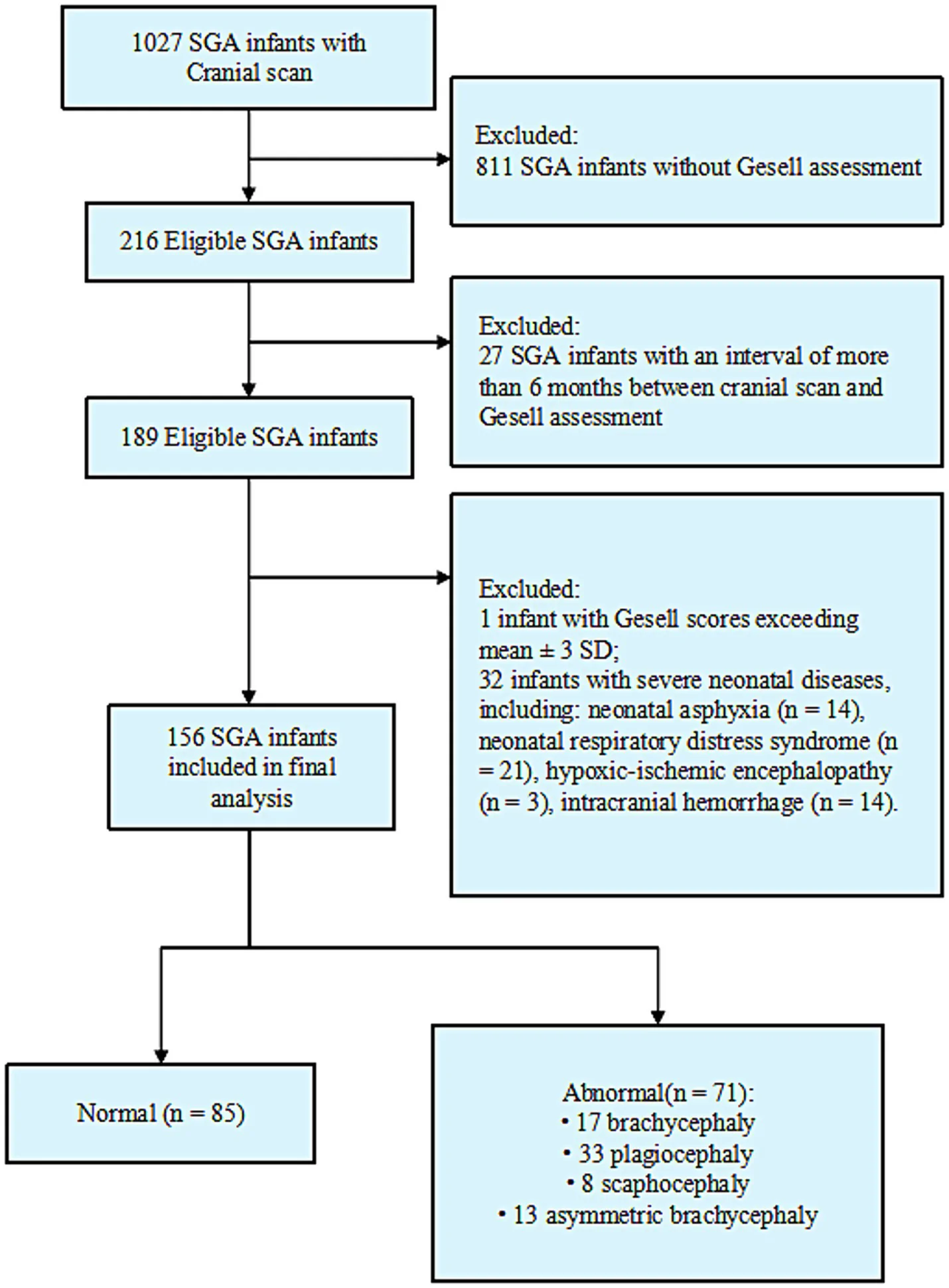
Flow diagram of participant selection. SGA small for gestational age, SD standard deviation.

The study was approved by the Institutional Review Board of Shanghai Children’s Hospital (No. 2025R150-E02) and was conducted in accordance with the Declaration of Helsinki. Written informed consent was obtained from the parents or legal guardians of all participants.

### Cranial morphology assessment

We captured cranial morphological parameters through the SmartSoc system, a 3D imaging platform employing computer vision to convert two-dimensional (2D) video into digital geometries. The hardware setup included a certified smartphone equipped with the CurveCapture O&P application, a patterned SmartSoc sleeve, a registration tag and anatomical landmarks (Figure 2). Data processing was conducted via the eBrace Global web portal. Trained technicians scanned all participants following a standardized protocol (Supplementary material 1). The acquired three-dimensional data were then analyzed using dedicated software to extract cranial measurements and morphological characteristics. Head shape was classified into five categories based on the cranial index (CI) and cranial vault asymmetry index (CVAI), as illustrated in Figure 3. Brachycephaly was defined as CI > 90, plagiocephaly as CVAI ≥ 3.5%, and scaphocephaly as CI ≤ 78. Infants meeting criteria for both brachycephaly and plagiocephaly were classified as asymmetric brachycephaly. Infants not meeting any of these criteria were classified as having normal head shape (16, 17).

**Figure 2:**
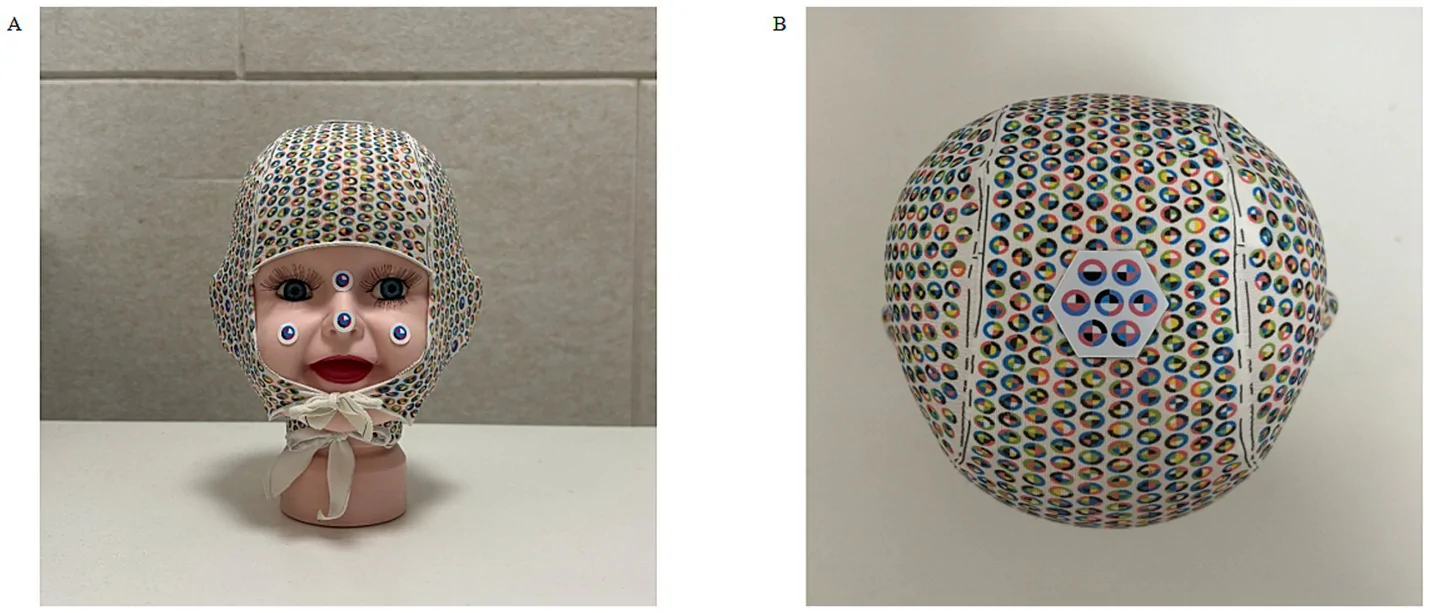
Anatomical landmarks and correct positioning of the measurement sleeve. **(A)** Illustration of sleeve donning and anatomical landmark placement. **(B)** Illustration of registration tag placement.

**Figure 3:**
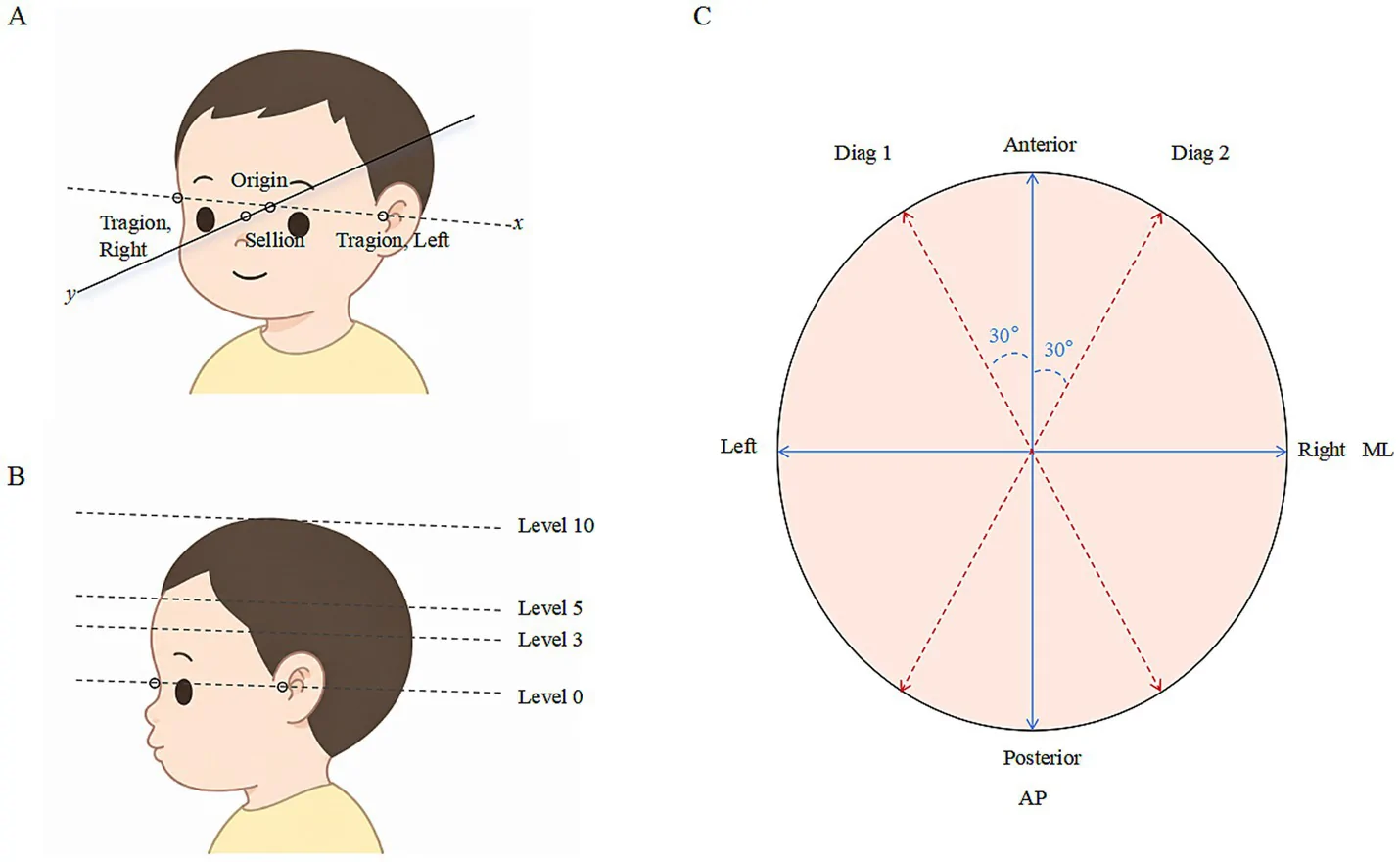
Cranial measurement procedures and index calculations. **(A)** Oblique frontal view showing landmark identification and construction of the cranial coordinate system. The sellion corresponds to the point of greatest concavity at the soft-tissue nasofrontal junction, whereas the tragia are located at the superior margins of the bilateral tragi. The midpoint between the two tragia serves as the coordinate origin. The transverse (X) axis passes through both tragia and the origin. The longitudinal (Y) axis extends from the origin toward the sellion. **(B)** Lateral view showing the axial reference system used for cranial assessment. The plane defined by the sellion and bilateral tragia was designated Level 0. Parallel sections were then positioned at regular intervals between this reference plane and the vertex, with the vertex corresponding to Level 10. Level 3 was selected for the cranial measurements used in the present analysis. **(C)** Superior view of the Level 3 cranial contour. Anteroposterior (AP) and mediolateral (ML) dimensions were measured along the corresponding orthogonal axes, and two oblique diameters (Diag1 and Diag2) were obtained at +30° and −30° relative to the AP axis. Cranial indices were derived as follows: cephalic index (CI) = ML/AP × 100; diagonal difference (Diff) = |Diag1 − Diag2|; and cranial vault asymmetry index (CVAI) = Diff/max(Diag1, Diag2) × 100. Abbreviations: AP anteroposterior diameter, ML mediolateral diameter, CI cephalic index, Diff diagonal difference, CVAI cranial vault asymmetry index.

### Gesell developmental schedules

Qualified assessors administered the Gesell Developmental Schedules (GDS) and generated scores for five domains: gross motor, fine motor, language, adaptive behavior, and personal-social behavior. For infants born preterm, developmental assessments were based on corrected age to account for prematurity. Results are expressed as Developmental Quotient (DQ) scores. Based on the scoring criteria, developmental delay was defined as a DQ ≤ 75 (18).

### Covariate selection

Based on previous literature, we included the following covariates obtained from medical records and parental questionnaires: infant sex, gestational age at birth, age at Gesell assessment, maternal education level, and pregnancy complications, including gestational hypertension, gestational diabetes mellitus, thyroid disorders, and hepatic dysfunction (19–21). Maternal education was retained as a three-level variable, with missing values treated as a separate category.

### Statistical analysis

For descriptive analyses, continuous variables are shown as mean ± SD when approximately normally distributed and as median (interquartile range [IQR]) when skewed. Categorical variables are expressed as number and percentage. Comparisons between groups used independent-samples t tests, with Welch’s correction when variances were unequal, Mann–Whitney U tests for non-normally distributed variables, and χ^2^ tests for categorical variables.

We used multiple linear regression to evaluate relationships between cranial morphology and Gesell DQ scores across five developmental domains. Two exposure specifications were examined: overall cranial deformation (normal vs. abnormal) and cranial subtype (normal, brachycephaly, plagiocephaly, scaphocephaly, and asymmetric brachycephaly), with the normal group as the reference. Each model was adjusted for infant sex, gestational age at birth, age at Gesell assessment, maternal education level, and pregnancy complications. Collinearity among covariates was evaluated using variance inflation factors (all VIF < 5). Residual distributions were examined per outcome domain using Shapiro–Wilk testing and Q–Q plot inspection, which indicated mild-to-moderate left skewness in four of the five domains (skewness range: −1.3 to −0.6). Although residual normality was violated in some models, the analytic sample size was considered sufficient for approximate inference based on the central limit theorem (22). HC3 heteroscedasticity-consistent standard errors were used to account for potential heteroscedasticity and non-normality of residuals, whereas conventional OLS standard errors were retained otherwise (23). Regression coefficients (β) and corresponding 95% confidence intervals are presented. To account for multiple testing, *p* values were corrected across all tests in each model using the Benjamini–Hochberg procedure to control the false discovery rate (FDR) (24, 25).

We performed four sensitivity analyses to examine the stability of the main results: (i) excluding infants born to mothers with pregnancy complications; (ii) restricting to infants with gestational age at birth ≥ 34 weeks; (iii) restricting to complete cases with no missing covariate data; and (iv) adjusting for birth-weight-for-gestational-age z-score (BWZ), calculated using the INTERGROWTH-21st sex- and gestational-age-specific standards. All analyses were conducted in R (version 4.4.1), and two-sided *p* < 0.05 was considered statistically significant.

## Results

### Baseline characteristics

Of the 1,027 infants who underwent cranial scanning, 156 met eligibility criteria and entered the final analysis: 85 had normal head shape, and 71 exhibited cranial deformation (Figure 1). Baseline demographic characteristics stratified by head shape are summarized in Table 1. Assessments were concentrated in early infancy: the median age was 6.36 months (IQR, 4.80–9.18) at cranial scanning and 6.90 months (IQR, 6.12–10.08) at Gesell assessment. No significant difference in sex, cranial and Gesell assessment age, maternal education level, or gestational complications was observed between infants with and without cranial deformation (all *p* > 0.05). Gestational age at birth was lower in infants with cranial deformation than in those with normal head shape (median, 37.00 vs. 37.57 weeks; *p* = 0.018). The distribution of gestational-age categories also differed between the groups (*p* = 0.011), primarily reflecting a higher proportion of infants born before 34 weeks in the cranial-deformation group (23.9% vs. 7.1%). Gestational age at birth was therefore adjusted for as a potential confounder in all subsequent analyses. However, no statistically significant between-group difference was detected in birth-weight-for-gestational-age percentile (median, 4.98 vs. 6.35; *p* = 0.381) or in the distribution of SGA severity (*p* = 0.413). Among the 71 infants with cranial deformation, plagiocephaly was the most common subtype (46.5%, *n* = 33), followed by brachycephaly (23.9%, *n* = 17) and asymmetric brachycephaly (18.3%, *n* = 13). Scaphocephaly was the least common subtype (11.3%, *n* = 8).

**Table 1:** Baseline characteristics of participants stratified by head-shape group.

Characteristic	Overall (*n* = 156)	Normal (*n* = 85)	Abnormal (*n* = 71)	*p* value
Sex, n (%)				0.964
Male	86 (55.1)	47 (55.3)	39 (54.9)	
Female	70 (44.9)	38 (44.7)	32 (45.1)	
Age at Gesell assessment (month), median [IQR]	6.90 [6.12, 10.08]	6.96 [6.12, 11.28]	6.84 [6.24, 9.12]	0.892
Age at cranial scan (month), median [IQR]	6.36 [4.80, 9.18]	6.00 [4.56, 9.00]	6.48 [5.64, 10.08]	0.064
Gestational age at birth (week), median [IQR]	37.21 [35.43, 39.14]	37.57 [36.29, 39.29]	37.00 [34.00, 39.00]	0.018
Gestational-age category at birth, n (%)				0.011
Term (≥ 37 weeks)	96 (61.5)	56 (65.9)	40 (56.3)	
Late preterm (34–36 weeks)	37 (23.7)	23 (27.1)	14 (19.7)	
Earlier preterm (<34 weeks)	23 (14.7)	6 (7.1)	17 (23.9)	
Birth-weight-for-gestational-age percentile, median [IQR]	5.21 [2.01, 8.14]	6.35 [2.25, 8.14]	4.98 [1.95, 8.01]	0.381
SGA severity, n (%)				0.413
<3rd percentile	54 (34.6)	27 (31.8)	27 (38.0)	
3rd - < 10th percentile	102 (65.4)	58 (68.2)	44 (62.0)	
Maternal education, n (%)^a^				0.820
Below bachelor’s degree	28 (18)	14 (16)	14 (20)	
Bachelor’s degree or above	80 (51)	42 (49)	38 (54)	
Missing	48 (31)	29 (34)	19 (27)	
Pregnancy complications, n (%)				0.244
Yes	31 (19.9)	14 (16.5)	17 (23.9)	
No	125 (80.1)	71 (83.5)	54 (76.1)	
Cranial deformity subtype, n (%)				
brachycephaly	17 (10.9)	NA	17 (23.9)	
plagiocephaly	33 (21.2)	NA	33 (46.5)	
scaphocephaly	8 (5.1)	NA	8 (11.3)	
asymmetric brachycephaly	13 (8.3)	NA	13 (18.3)	

Data are presented as n (%) or median (interquartile range). Continuous variables compared with two-sample t test or Mann–Whitney U test as appropriate; categorical variables compared with χ^2^ test or Fisher exact test as appropriate. IQR interquartile range. NA, not applicable.

a Missing values were shown descriptively but excluded from the between-group test for maternal education.

To evaluate potential selection bias, we compared baseline characteristics between infants included in the final analysis (*n* = 156) and those who were not included (*n* = 871) among the 1,027 SGA infants who underwent cranial scanning during the study period (Supplementary Table 1). Compared with the non-included group, included infants had lower gestational age at birth, older corrected age at cranial scanning, and a higher prevalence of maternal pregnancy complications. Sex distribution did not differ significantly between the groups, whereas maternal education differed significantly. The proportion of abnormal cranial morphology was also similar between the included and non-included groups (45.5% vs. 42.1%; *p* = 0.432). Infant sex, gestational age at birth, age at Gesell assessment, maternal education, and pregnancy complications were retained in the subsequent multivariable regression models.

### Associations between cranial deformation and neurodevelopmental outcomes

#### Overall associations between cranial deformation and neurodevelopment

In multiple linear regression models adjusted for infant sex, gestational age at birth, Gesell assessment age, maternal education level, and pregnancy complications, the presence of cranial deformation was significantly associated with lower developmental quotient scores in the fine motor domain (*β* = −4.97, 95% CI: −8.71 to −1.24; *p* = 0.009, *P*_FDR_ = 0.044) and the adaptive behavior domain (*β* = −4.91, 95% CI: −8.96 to −0.86; *p* = 0.018, *P*_FDR_ = 0.044), compared with infants with normal head shape (Table 2). Notably, these associations remained statistically significant after applying False Discovery Rate (*P*_FDR_ < 0.05). A marginal association was also observed in the gross motor domain (*β* = −3.82, 95% CI: −7.93 to 0.30; *p* = 0.069).

**Table 2:** Multivariable regression of head-shape abnormality on Gesell developmental quotients.

Developmental domain	*β* (95% CI)	*p* value	FDR-adjusted *p* value
Gross motor DQ	−3.82 (−7.93 to 0.30)	0.069	0.115
Fine motor DQ	−4.97 (−8.71 to −1.24)	0.009	0.044*
Adaptive DQ	−4.91 (−8.96 to −0.86)	0.018	0.044*
Language DQ	−2.87 (−6.29 to 0.55)	0.099	0.124
Personal-social DQ	−2.46 (−5.67 to 0.74)	0.131	0.131

Each domain was analyzed using a separate multivariable linear regression model, with head-shape abnormality as the exposure and normal head shape as the reference. Models were adjusted for sex, age at Gesell assessment, gestational age at birth, pregnancy complications, and maternal education. FDR-adjusted *p* values were calculated using the Benjamini–Hochberg procedure across the five domains. β, regression coefficient in DQ points; CI, confidence interval; DQ, developmental quotient; FDR, false discovery rate, *p FDR < 0.05.

#### Subtype-specific associations

Subtype-specific regression analyses (Table 3) suggested similar negative associations for brachycephaly and plagiocephaly in the fine motor and adaptive behavior domains before FDR correction. However, none of the subtype-specific associations remained significant after FDR correction, and no clear associations were observed for scaphocephaly or asymmetric brachycephaly.

**Table 3:** Multivariable regression of head-shape subtypes on Gesell developmental quotients.

Developmental domain	Subtype	*β* (95% CI)	*p* value	FDR-adjusted *p* value
Gross motor DQ	Brachycephaly	−4.47 (−12.92 to 3.98)	0.297	0.514
	Plagiocephaly	−3.46 (−8.65 to 1.72)	0.189	0.471
	Scaphocephaly	−4.88 (−13.88 to 4.11)	0.285	0.514
	Asymmetric brachycephaly	−3.03 (−12.22 to 6.17)	0.516	0.737
Fine motor DQ	Brachycephaly	−5.91 (−10.79 to −1.02)	0.018	0.126
	Plagiocephaly	−5.76 (−10.79 to −0.72)	0.025	0.126
	Scaphocephaly	−4.82 (−14.14 to 4.50)	0.308	0.514
	Asymmetric brachycephaly	−1.15 (−7.99 to 5.68)	0.740	0.871
Adaptive DQ	Brachycephaly	−6.60 (−12.12 to −1.09)	0.019	0.126
	Plagiocephaly	−7.27 (−12.74 to −1.81)	0.009	0.126
	Scaphocephaly	0.34 (−9.00 to 9.67)	0.943	0.943
	Asymmetric brachycephaly	0.63 (−5.48 to 6.75)	0.838	0.882
Language DQ	Brachycephaly	−5.56 (−11.17 to 0.06)	0.052	0.210
	Plagiocephaly	−3.73 (−8.00 to 0.53)	0.086	0.286
	Scaphocephaly	3.15 (−4.54 to 10.84)	0.420	0.646
	Asymmetric brachycephaly	−1.34 (−7.89 to 5.21)	0.686	0.858
Personal-social DQ	Brachycephaly	−4.07 (−9.75 to 1.60)	0.158	0.453
	Plagiocephaly	−2.59 (−6.82 to 1.64)	0.228	0.507
	Scaphocephaly	−0.84 (−7.88 to 6.19)	0.813	0.882
	Asymmetric brachycephaly	−1.05 (−5.97 to 3.87)	0.674	0.858

Each domain was analyzed using a separate multivariable linear regression model with normal head shape as the reference. Models were adjusted for sex, age at Gesell assessment, gestational age at birth, pregnancy complications, and maternal education. FDR-adjusted p values were calculated using the Benjamini–Hochberg procedure across all 20 subtype-domain contrasts. β, regression coefficient in DQ points; CI, confidence interval; DQ, developmental quotient; FDR, false discovery rate.

#### Associations between continuous cranial indices and developmental quotients

Multivariable linear regressions were used to assess CI and CVAI as continuous predictors of neurodevelopment (Table 4). A higher CI was associated with lower language DQ, though this did not remain significant after FDR correction. No other domains showed significant linear associations with CI. Regarding asymmetry, higher CVAI exhibited marginal negative trends with fine motor DQ and adaptive DQ. However, these potential correlations did not reach statistical significance following multiple comparison adjustments.

**Table 4:** Multivariable regression of cephalic index and cranial vault asymmetry index on Gesell developmental quotients.

Developmental domain	Predictor	*β* (95% CI)	*p* value	FDR-adjusted *p* value
Gross motor DQ	CI	−0.07 (−0.51 to 0.38)	0.775	0.862
	CVAI	−0.55 (−1.53 to 0.44)	0.274	0.547
Fine motor DQ	CI	−0.09 (−0.47 to 0.29)	0.634	0.793
	CVAI	−0.86 (−1.74 to 0.02)	0.055	0.215
Adaptive DQ	CI	−0.17 (−0.56 to 0.23)	0.402	0.574
	CVAI	−0.84 (−1.74 to 0.05)	0.064	0.215
Language DQ	CI	−0.37 (−0.69 to −0.04)	0.027	0.215
	CVAI	−0.05 (−0.79 to 0.69)	0.898	0.898
Personal-social DQ	CI	−0.24 (−0.54 to 0.06)	0.123	0.307
	CVAI	−0.30 (−0.93 to 0.33)	0.348	0.574

Each domain was analyzed using a separate multivariable linear regression model, with cephalic index and cranial vault asymmetry index entered jointly as continuous predictors. Models were adjusted for sex, age at Gesell assessment, gestational age at birth, pregnancy complications, and maternal education. β represents the change in DQ score per 1-point increase in the corresponding cranial index. FDR-adjusted *p* values were calculated using the Benjamini–Hochberg procedure across all 10 tests. β, regression coefficient; CI, cephalic index; 95% CI, 95% confidence interval; CVAI cranial vault asymmetry index; DQ, developmental quotient; FDR, false discovery rate.

#### Prevalence of developmental delay across head-shape groups

We further evaluated the prevalence of developmental delay (DQ ≤ 75) relative to head shape (Table 5). The abnormal head-shape group showed a higher observed proportion of gross motor delay compared to the normal group (12.7% vs. 3.5%, crude OR = 3.97, 95% CI: 1.03–15.27, *p* = 0.033), but this association did not remain significant after FDR correction. Numerically higher delay rates were also observed across the remaining four domains in the abnormal group, including fine motor (9.9% vs. 5.9%), adaptive behavior (14.1% vs. 5.9%), language (14.1% vs. 8.2%), and personal-social domains (9.9% vs. 7.1%), although none reached statistical significance (*p* > 0.05).

**Table 5:** Prevalence of developmental delay by head-shape group.

Developmental domain	Normal (*n* = 85)	Abnormal (*n* = 71)	*p* value	Crude OR	95% CI	FDR-adjusted *p* value
Gross motor DQ	3/85 (3.5)	9/71 (12.7)	0.033	3.97	1.03–15.27	0.164
Fine motor DQ	5/85 (5.9)	7/71 (9.9)	0.353	1.75	0.53–5.77	0.442
Adaptive DQ	5/85 (5.9)	10/71 (14.1)	0.084	2.62	0.85–8.07	0.209
Language DQ	7/85 (8.2)	10/71 (14.1)	0.243	1.83	0.66–5.08	0.405
Personal-social DQ	6/85 (7.1)	7/71 (9.9)	0.529	1.44	0.46–4.50	0.529

Developmental delay was defined as Gesell developmental quotient (DQ) ≤ 75. Crude odds ratios (OR) with 95% confidence intervals (CI) are shown. Pearson χ^2^ test was used for all five domains (minimum expected cell count ≥ 5). OR > 1 indicates higher odds of delay in the abnormal group relative to the normal group. FDR-adjusted *p* values were calculated using the Benjamini–Hochberg procedure across the five domains. DQ, developmental quotient.

### Sensitivity analyses

Sensitivity analyses were conducted to examine whether the observed associations between cranial morphology and neurodevelopmental outcomes remained consistent across different analytical scenarios. In the overall cranial morphology model, findings were broadly consistent across all four scenarios. After excluding infants born to mothers with pregnancy complications (*n* = 125), the negative associations with fine motor (*β* = −6.34, 95% CI: −10.81 to −1.87, *P*_FDR_ = 0.020) and adaptive behavior quotients (*β* = −6.22, 95% CI: −10.79 to −1.65, *P*_FDR_ = 0.020) remained statistically significant after FDR correction, with effect sizes modestly larger than in the primary analysis. Gross motor DQ also showed a nominally significant inverse association, but this association did not remain statistically significant after FDR correction (*β* = −4.83, *p* = 0.034, *P*_FDR_ = 0.057). When the sample was restricted to late-preterm and term infants (GA ≥ 34 weeks, *n* = 133), the negative associations with fine motor and adaptive behavior quotients remained comparable to those in the primary analysis and likewise survived FDR correction. When restricted to complete cases with no missing covariates (*n* = 108), point estimates for fine motor and adaptive behavior quotients remained negative and directionally consistent with the primary analysis, but did not survive FDR correction. Additional adjustment for BWZ did not materially alter the results, with the associations with lower fine motor and adaptive behavior DQ remaining significant after FDR correction (both *P_FDR_* = 0.047). Language and personal-social quotients showed no significant associations across any of the four scenarios after FDR correction.

In the subtype analyses, the pattern of negative associations between brachycephaly and plagiocephaly with fine motor and adaptive behavior quotients was reproduced directionally across all four scenarios, though no subtype-specific association survived global FDR correction. Scaphocephaly and asymmetric brachycephaly showed no consistent associations in any scenario. Full results from all sensitivity analyses are presented in Supplementary Tables 2, 3.

## Discussion

To our knowledge, few studies have specifically examined the associations between positional cranial deformities and domain-specific neurodevelopmental outcomes in SGA infants. After adjusting for sex, gestational age at birth, age at assessment, maternal education, and pregnancy complications, we found that the presence of cranial deformity was significantly associated with reduced developmental quotients in the fine motor and adaptive behavior domains.

Our findings align with the overall trends observed in the general population while highlighting certain distinctions. A systematic review identified the motor domain as the most consistently affected area in high-quality studies, noting that these associations are more frequent in infants under 24 months (26). While previous research has predominantly reported delays in gross motor development (26–28), our study found that cranial abnormalities were more clearly associated with decreased fine motor scores. Gross motor scores were also numerically lower in infants with cranial deformity; however, the between-group difference was not statistically significant. This primary finding regarding fine motor and adaptive behavior is consistent with the 36-month follow-up results from Collett et al. in 2013, which showed that infants with positional plagiocephaly scored significantly lower in these domains (29). This finding may be interpreted in the context of previous studies on neurodevelopment in children born SGA. Differences in fine-motor control have been reported in SGA infants, and a community-based cohort showed lower fine motor scores with less marked differences in gross motor performance (30, 31). Fine motor and adaptive behaviors require hand-eye coordination, object manipulation, and active exploration; these skills may therefore be more susceptible to limited postural variation or reduced sensorimotor experience (32). As these experiences were not directly assessed in our cohort, this explanation should be interpreted with caution.

These domain-specific differences should also be interpreted in light of the early assessment age (median, 6.90 months). At this stage, GDS scores describe current developmental abilities and provide a baseline for follow-up. The approximately 5-point lower adjusted fine motor and adaptive DQ scores therefore support closer attention to these domains in SGA infants with cranial deformation, with individual interpretation guided by the overall developmental profile and clinical findings. Longitudinal studies have linked early Gesell performance to selected later nonverbal and visuomotor abilities (33, 34), suggesting that early developmental findings may have relevance beyond the time of assessment. Although it remains unclear whether the fine motor and adaptive DQ differences observed in our cohort persist or are associated with later functional difficulties, our findings suggest that cranial morphology may serve as an early observable indicator to help identify SGA infants who warrant closer developmental assessment and follow-up, particularly in these two domains.

Furthermore, this study found no significant linear associations between continuous cranial indices and developmental scores after FDR correction; associations were observed only when cranial morphology was analyzed categorically. Lynch et al. observed that the cranial-deformity severity was not clearly associated with developmental disorder diagnoses by age seven in a cohort of 9,909 infants, suggesting that persistent delay does not occur in most affected infants (16). Collett et al. also reported in a school-age follow-up that children with moderate to severe deformities scored lower in IQ and academic achievement, whereas those with mild deformities showed no significant differences from controls (9). These observations are partly in line with our findings and support the possibility that developmental vulnerability may emerge only after cranial deviation exceeds a certain threshold, rather than increasing proportionally with deformity severity. However, this hypothesis requires further validation in larger cohorts.

Exploratory analysis suggests that the observed associations may be related to both brachycephaly and plagiocephaly. Although specific subtype-domain correlations did not maintain significance under FDR correction, the similarity in their effect sizes provides a basis for discussing potential biological mechanisms. Importantly, these findings should not be interpreted as evidence that cranial deformation itself causes poorer developmental performance. Rather, cranial deformation and developmental performance may partly reflect shared underlying vulnerabilities. Research by Zielińska et al. proposed that cranial abnormalities are not isolated anatomical defects but may be associated with broader psychomotor developmental signals (35). Such shared vulnerabilities may include neuromotor immaturity, altered muscle tone, postural preference, and potentially other neonatal or developmental factors. Considering the evidence regarding muscle tone abnormalities as a comorbidity (8), these factors could contribute both to persistent head positioning and to reduced spontaneous movement or postural control, potentially limiting sensorimotor experiences (35). Because both fine motor skills and adaptive behavior rely heavily on hand-eye coordination and object exploration, the lack of postural variety may render these domains particularly susceptible to developmental differences. However, these mechanisms were not directly assessed in the present study and therefore remain hypothetical.

In contrast, scaphocephaly and asymmetric brachycephaly did not show consistent trends or significant associations in our sample. Given the limited number of cases within these specific subgroups, the current data are insufficient to draw reliable conclusions.

Beyond the analysis of continuous DQ scores, the categorical analysis of clinically defined developmental delay (DQ ≤ 75; Table 5) provided a complementary threshold-based perspective. Infants with cranial deformity had a higher observed prevalence of gross motor delay than those with normal cranial morphology, although this association did not remain significant after FDR correction. Higher proportions of delay were also observed in the adaptive and language domains, but these differences were not statistically significant. Taken together with the continuous DQ analyses, these findings suggest that cranial deformity in SGA infants may be associated with less favorable developmental profiles, particularly in fine motor, adaptive, and possibly gross motor domains.

The consistency in effect direction and magnitude across all four sensitivity analyses supports the robustness of the primary findings. FDR-adjusted significance was retained after excluding obstetric complications and after restricting to GA ≥ 34 weeks, suggesting that obstetric complications and early preterm birth did not confound the observed associations. In the complete-case analysis, point estimates remained closely comparable to the primary analysis, and the loss of FDR-adjusted significance most plausibly reflects reduced statistical power from the smaller sample size rather than a genuine weakening of the associations. Notably, brachycephaly and plagiocephaly consistently showed negative point estimates for fine motor and adaptive behavior scores across multiple sensitivity analyses, suggesting these two subtypes may have contributed to the overall pattern of associations.

Despite these findings, several limitations of our study warrant consideration. First, the cross-sectional design inherently restricts our ability to draw causal links between cranial changes and developmental trajectories. Second, although cranial scanning was routinely offered during SGA follow-up, selection bias cannot be excluded because this was a clinical follow-up cohort rather than a population-based sample. The analytic sample was enriched for infants with greater perinatal risk, possibly reflecting referral and follow-up patterns at our tertiary hospital. Consequently, the generalizability of our findings to lower-risk SGA populations may be limited, and the observed proportion of cranial deformation should not be interpreted as a prevalence estimate for the broader SGA population. Nevertheless, the similar proportions of abnormal cranial morphology in the included and non-included groups provide no evidence of additional preferential inclusion based on cranial morphology after scanning. Multicenter prospective studies recruiting consecutive SGA infants irrespective of clinical concern are needed to confirm the findings across more diverse clinical populations.

Third, although infants with major neonatal conditions known to affect neurodevelopment were excluded, detailed characterization of neonatal clinical history—including NICU admission, duration of hospitalization, respiratory support, feeding difficulties, and other postnatal complications—was limited. Residual confounding related to neonatal morbidity therefore cannot be entirely excluded. Future prospective studies should systematically collect and account for these clinical characteristics. Fourth, although the overall sample size was adequate for the primary analyses, subgroup sizes for individual deformation subtypes were limited, which constrained statistical power for subtype-specific comparisons and warrants caution in interpreting those results. Finally, although infants with known major conditions affecting neurodevelopment were excluded, systematic neuroimaging was not performed in all participants. Therefore, subtle intracranial abnormalities, neuromotor dysfunction, or other unrecognized systemic conditions cannot be excluded as shared factors underlying both cranial deformation and developmental performance. Longitudinal developmental outcomes were not examined, so the persistence of these associations remains unclear.

This study suggests that cranial deformation in SGA infants is associated with less favorable developmental profiles, particularly in the fine motor and adaptive behavior domains. Although exploratory subtype analyses suggested nominal associations of both brachycephaly and plagiocephaly with lower fine motor and adaptive behavior scores, none of these subtype-specific associations survived FDR correction and should therefore be interpreted with caution. These findings highlight the potential value of integrating cranial shape assessment into routine SGA surveillance, as cranial morphology may provide an accessible clinical indicator for identifying infants who could benefit from closer developmental monitoring and individualized guidance on postural variability and sensorimotor engagement. Multicenter prospective studies are needed to validate these associations and clarify their long-term developmental implications.

## Data Availability

Owing to privacy and ethical considerations related to clinical information from infants and young children, the data used in this study are not openly accessible. Anonymized data may be obtained from the corresponding author upon reasonable request, subject to applicable ethical approvals.
