# Supplementary material for: Positional cranial deformation and domain-specific neurodevelopment in small-for-gestational-age infants: a cross-sectional study
Source: Front Public Health. 2026 Oct 27;14:1936897. doi: 10.3389/fpubh.2026.1936897 (PMC13748429; doi:10.3389/fpubh.2026.1936897)
Supplement: Supplementary file 1 [file Supplementary_file_1.DOCX]

**Supplementary Material S1: SmartSoc 3D Cranial Imaging Protocol**

**1. Scanning Preparation**

1.1 Sleeve Selection and Application

An appropriately sized SmartSoc cranial sleeve was selected based on head circumference measured at the supraorbital margin. The sleeve was carefully applied to ensure complete, wrinkle-free coverage of the craniofacial region without excessive tension and was secured submentally.

**1.2 Anatomical Landmark Identification**

Adhesive markers were precisely positioned at standardized anatomical locations:

- Sellion:the deepest point of the soft-tissue nasofrontal angle, located between the frontal slope and the proximal nasal bridge
- Pronasale: The most anterior projection of the nasal apex
- Tragion: The superior margin of the tragus.

1.3 Registration Markers and Accessory Positioning

A registration tag (hexagonal fiducial marker) was adhered to the vertex region overlying the sleeve. Ear covers were then positioned to encompass the auricular regions bilaterally, with edges aligned to auricular contours.

**2. Three-Dimensional Image Acquisition**

2.1 System Setup and Authentication

The operator accessed the CurveCapture O&P application on a certified smartphone device.

2.2 Image Capture Protocol

The acquisition process followed a standardized three-region scanning sequence:

- Registration Capture: The registration tag was aligned with the on-screen hexagonal template. Camera angulation was systematically adjusted until automated registration confirmation.
- Cranial Surface Scanning: The patient maintained an upright seated position with neutral head posture. Systematic scanning encompassed:
  - Superior region (dome): Camera positioned at approximately 45° downward angle circumferentially around the cranial vault.
  - Anterior region (face): Image capture at eye level from the sagittal plane and bilateral oblique angles (±15°-20°).
  - Posterolateral regions: Camera positioned 15° above horizontal, capturing temporal and occipital surfaces.

**3. Data Processing and Measurement**

3.1 Automated Image Analysis

Upon completion, scan data were automatically transmitted to the secure eBrace Global cloud-based platform via secure wireless network connection. Within the portal, the three-dimensional models were processed using the Measurement and Comparison Utility (MCU) software. Each model was aligned to a standardized coordinate system defined by the sellion and bilateral tragia. The midpoint between the two tragia served as the origin. The transverse (X) axis passed through both tragia and the origin, while the longitudinal (Y) axis extended from the origin toward the sellion. The plane defined by these three landmarks served as the reference plane (Level 0).

3.2 Morphometric Assessment

Cranial measurements (including but not limited to cranial diameter asymmetry, antero-posterior length, and transverse width) were automatically extracted based on the standardized alignment. All measurements were performed by a single trained operator to improve measurement consistency.
