# Supplementary material for: Positional cranial deformation and domain-specific neurodevelopment in small-for-gestational-age infants: a cross-sectional study
Source: Front Public Health. 2026 Oct 27;14:1936897. doi: 10.3389/fpubh.2026.1936897 (PMC13748429; doi:10.3389/fpubh.2026.1936897)
Supplement: Supplementary file 2 [file Table_1.DOCX]

**Supplementary Table 1 Comparison of SGA Infants Included and Not Included in the Final Analysis**

| **Characteristic** | **Not included**  **(n = 871)** | **Included**  **(n = 156)** | ***P* value** |
| --- | --- | --- | --- |
| Sex, n (%) |  |  | 0.733 |
| Male | 493 (56.6) | 86 (55.1) |  |
| Female | 378 (43.4) | 70 (44.9) |  |
| Age at cranial scan (month, corrected)^a^, median [IQR] | 4.44 [3.48, 6.12] | 6.36 [4.80, 9.18] | <0.001 |
| Gestational age at birth (week), median [IQR] | 39.14 [38.00, 40.00] | 37.21 [35.43, 39.14] | <0.001 |
| Maternal education, n (%)^b^ |  |  | 0.023 |
| Below bachelor's degree | 50 (5.7) | 28 (17.9) |  |
| Bachelor's degree or above | 262 (30.1) | 80 (51.3) |  |
| Missing | 559 (64.2) | 48 (30.8) |  |
| Pregnancy complications, n (%) |  |  | <0.001 |
| None | 814 (93.5) | 125 (80.1) |  |
| Present | 57 (6.5) | 31 (19.9) |  |
| Cranial morphology, n (%) |  |  | 0.432 |
| Normal | 504 (57.9) | 85 (54.5) |  |
| Abnormal | 367 (42.1) | 71 (45.5) |  |

*Continuous variables were compared using the Mann–Whitney U test as residuals deviated from normality (Shapiro-Wilk P < 0.05). Categorical variables were compared using Pearson's χ² test. ^a^Corrected age was used for preterm infants (gestational age < 37 weeks), chronological age was used for term infants. ^b^Missing values were shown descriptively but excluded from the between-group test for maternal education.*

**Supplementary Table 2 Sensitivity analyses for the association between overall cranial deformity and Gesell developmental quotients**

| **Developmental domain** | **N** | ***β* (95% CI)** | **P value** | **FDR-adjusted *P* value** |
| --- | --- | --- | --- | --- |
| *Excluding pregnancy complications (n = 125)* | | | | |
| Gross Motor | 125 | -4.83 (-9.29 to -0.37) | 0.034 | 0.057 |
| Fine Motor | 125 | -6.34 (-10.81 to -1.87) | 0.006 | 0.020* |
| Adaptive Behavior | 125 | -6.22 (-10.79 to -1.65) | 0.008 | 0.020* |
| Language | 125 | -2.44 (-6.41 to 1.54) | 0.228 | 0.284 |
| Personal-Social | 125 | -1.85 (-5.62 to 1.92) | 0.333 | 0.333 |
| *GA ≥ 34 weeks (n = 133)* | | | | |
| Gross Motor | 133 | -3.51 (-7.99 to 0.96) | 0.123 | 0.154 |
| Fine Motor | 133 | -5.29 (-9.48 to -1.09) | 0.014 | 0.047* |
| Adaptive Behavior | 133 | -5.37 (-9.84 to -0.90) | 0.019 | 0.047* |
| Language | 133 | -3.17 (-6.86 to 0.52) | 0.092 | 0.153 |
| Personal-Social | 133 | -2.20 (-5.75 to 1.35) | 0.223 | 0.223 |
| *Complete cases (n = 108)* | | | | |
| Gross Motor | 108 | -2.53 (-7.58 to 2.52) | 0.323 | 0.323 |
| Fine Motor | 108 | -4.91 (-9.52 to -0.29) | 0.037 | 0.116 |
| Adaptive Behavior | 108 | -4.51 (-9.39 to 0.37) | 0.07 | 0.116 |
| Language | 108 | -3.89 (-8.00 to 0.21) | 0.062 | 0.116 |
| Personal-Social | 108 | -2.11 (-6.20 to 1.99) | 0.31 | 0.323 |
| *Additional adjustment for BWZ (n = 156)* | | | | |
| Gross Motor | 156 | -3.75 (−7.88 to 0.39) | 0.075 | 0.125 |
| Fine Motor | 156 | -4.95 (−8.70 to −1.21) | 0.01 | 0.047* |
| Adaptive Behavior | 156 | -4.89 (−8.94 to −0.83) | 0.019 | 0.047* |
| Language | 156 | -2.75 (−6.12 to 0.63) | 0.11 | 0.131 |
| Personal-Social | 156 | -2.50 (−5.75 to 0.75) | 0.131 | 0.131 |

*Each domain was analyzed using a separate multivariable linear regression model, with normal head shape as the reference. Models were adjusted for sex, age at Gesell assessment, gestational age at birth, pregnancy complications, and maternal education where applicable. FDR-adjusted P values were calculated using the Benjamini–Hochberg procedure across the five domains. β regression coefficient in DQ points, CI confidence interval, DQ, developmental quotient, FDR, false discovery rate. GA gestational age at birth. BWZ, birth-weight-for-gestational-age z-score.*P FDR < 0.05.*

**Supplementary Table 3 Sensitivity analyses for the association between cranial deformity subtypes and Gesell developmental quotients**

| **Cranial deformity subtype** | **Domain** | **N** | ***β* (95% CI)** | ***P* value** | **FDR-adjusted *P* value** |
| --- | --- | --- | --- | --- | --- |
| *Excluding pregnancy complications (n = 125)* | | | | | |
| Brachycephaly | Gross Motor | 125 | -4.73 (-14.89 to 5.43) | 0.358 | 0.604 |
|  | Fine Motor | 125 | -8.46 (-14.47 to -2.45) | 0.006 | 0.051 |
|  | Adaptive Behavior | 125 | -8.50 (-14.59 to -2.42) | 0.007 | 0.051 |
|  | Language | 125 | -8.65 (-15.52 to -1.79) | 0.014 | 0.07 |
|  | Personal-Social | 125 | -2.42 (-8.96 to 4.12) | 0.466 | 0.665 |
| Plagiocephaly | Gross Motor | 125 | -4.67 (-10.26 to 0.92) | 0.101 | 0.336 |
|  | Fine Motor | 125 | -6.82 (-12.66 to -0.97) | 0.023 | 0.091 |
|  | Adaptive Behavior | 125 | -8.32 (-14.39 to -2.25) | 0.008 | 0.051 |
|  | Language | 125 | -3.03 (-7.75 to 1.70) | 0.207 | 0.518 |
|  | Personal-Social | 125 | -2.27 (-7.18 to 2.65) | 0.362 | 0.604 |
| Scaphocephaly | Gross Motor | 125 | -7.24 (-19.33 to 4.84) | 0.238 | 0.528 |
|  | Fine Motor | 125 | -8.39 (-20.52 to 3.73) | 0.173 | 0.494 |
|  | Adaptive Behavior | 125 | -2.02 (-15.43 to 11.39) | 0.766 | 0.851 |
|  | Language | 125 | 4.21 (-4.77 to 13.20) | 0.355 | 0.604 |
|  | Personal-Social | 125 | -2.86 (-11.73 to 6.00) | 0.524 | 0.698 |
| Asymmetric brachycephaly | Gross Motor | 125 | -3.59 (-12.71 to 5.54) | 0.438 | 0.665 |
|  | Fine Motor | 125 | -0.41 (-9.06 to 8.24) | 0.926 | 0.926 |
|  | Adaptive Behavior | 125 | 0.55 (-7.37 to 8.47) | 0.89 | 0.926 |
|  | Language | 125 | 2.11 (-5.33 to 9.55) | 0.576 | 0.72 |
|  | Personal-Social | 125 | 1.12 (-4.67 to 6.92) | 0.702 | 0.825 |
| *GA ≥ 34 weeks (n = 133)* | | | | | |
| Brachycephaly | Gross Motor | 133 | -2.98 (-11.27 to 5.30) | 0.477 | 0.682 |
|  | Fine Motor | 133 | -5.51 (-11.03 to 0.01) | 0.05 | 0.31 |
|  | Adaptive Behavior | 133 | -5.29 (-10.96 to 0.39) | 0.067 | 0.31 |
|  | Language | 133 | -5.31 (-12.11 to 1.49) | 0.125 | 0.416 |
|  | Personal-Social | 133 | -2.20 (-8.08 to 3.68) | 0.46 | 0.682 |
| Plagiocephaly | Gross Motor | 133 | -3.64 (-9.49 to 2.22) | 0.221 | 0.575 |
|  | Fine Motor | 133 | -6.25 (-11.86 to -0.63) | 0.029 | 0.294 |
|  | Adaptive Behavior | 133 | -8.07 (-13.97 to -2.16) | 0.008 | 0.156 |
|  | Language | 133 | -4.13 (-8.72 to 0.46) | 0.078 | 0.31 |
|  | Personal-Social | 133 | -2.83 (-7.47 to 1.81) | 0.23 | 0.575 |
| Scaphocephaly | Gross Motor | 133 | -4.89 (-14.71 to 4.94) | 0.327 | 0.654 |
|  | Fine Motor | 133 | -4.98 (-14.63 to 4.67) | 0.309 | 0.654 |
|  | Adaptive Behavior | 133 | 0.11 (-9.58 to 9.80) | 0.982 | 0.982 |
|  | Language | 133 | 3.47 (-4.37 to 11.31) | 0.383 | 0.681 |
|  | Personal-Social | 133 | -0.56 (-8.16 to 7.04) | 0.884 | 0.948 |
| Asymmetric brachycephaly | Gross Motor | 133 | -2.20 (-11.73 to 7.33) | 0.649 | 0.854 |
|  | Fine Motor | 133 | -1.20 (-10.88 to 8.48) | 0.807 | 0.948 |
|  | Adaptive Behavior | 133 | -0.50 (-8.35 to 7.35) | 0.9 | 0.948 |
|  | Language | 133 | -3.49 (-11.81 to 4.84) | 0.408 | 0.681 |
|  | Personal-Social | 133 | -1.43 (-8.35 to 5.49) | 0.683 | 0.854 |
| *Complete cases (n = 108)* | | | | | |
| Brachycephaly | Gross Motor | 108 | -1.84 (-11.17 to 7.49) | 0.697 | 0.82 |
|  | Fine Motor | 108 | -6.06 (-11.06 to -1.05) | 0.018 | 0.252 |
|  | Adaptive Behavior | 108 | -5.67 (-11.56 to 0.22) | 0.059 | 0.295 |
|  | Language | 108 | -3.14 (-9.71 to 3.42) | 0.344 | 0.711 |
|  | Personal-Social | 108 | -2.62 (-9.21 to 3.98) | 0.433 | 0.711 |
| Plagiocephaly | Gross Motor | 108 | -2.58 (-9.42 to 4.25) | 0.455 | 0.711 |
|  | Fine Motor | 108 | -4.79 (-11.14 to 1.56) | 0.137 | 0.55 |
|  | Adaptive Behavior | 108 | -6.73 (-13.40 to -0.05) | 0.048 | 0.295 |
|  | Language | 108 | -6.05 (-11.34 to -0.77) | 0.025 | 0.252 |
|  | Personal-Social | 108 | -2.43 (-8.11 to 3.25) | 0.399 | 0.711 |
| Scaphocephaly | Gross Motor | 108 | -2.74 (-9.45 to 3.97) | 0.42 | 0.711 |
|  | Fine Motor | 108 | -4.83 (-18.90 to 9.23) | 0.497 | 0.711 |
|  | Adaptive Behavior | 108 | -0.06 (-13.77 to 13.64) | 0.993 | 0.993 |
|  | Language | 108 | 2.20 (-6.91 to 11.31) | 0.633 | 0.791 |
|  | Personal-Social | 108 | 0.58 (-9.18 to 10.33) | 0.907 | 0.993 |
| Asymmetric brachycephaly | Gross Motor | 108 | -3.38 (-16.81 to 10.04) | 0.618 | 0.791 |
|  | Fine Motor | 108 | -3.31 (-12.96 to 6.34) | 0.498 | 0.711 |
|  | Adaptive Behavior | 108 | 0.08 (-7.09 to 7.25) | 0.983 | 0.993 |
|  | Language | 108 | -4.51 (-12.60 to 3.57) | 0.271 | 0.711 |
|  | Personal-Social | 108 | -2.87 (-8.84 to 3.09) | 0.342 | 0.711 |
| *Additional adjustment for BWZ (n = 156)* | | | | | |
| Brachycephaly | Gross Motor | 156 | -4.62 (-13.13 to 3.88) | 0.284 | 0.502 |
|  | Fine Motor | 156 | -5.95 (-10.86 to -1.05) | 0.018 | 0.119 |
|  | Adaptive Behavior | 156 | -6.70 (-12.22 to -1.17) | 0.018 | 0.119 |
|  | Language | 156 | -5.80 (-11.35 to -0.26) | 0.040 | 0.162 |
|  | Personal-Social | 156 | -4.08 (-9.78 to 1.63) | 0.160 | 0.458 |
| Plagiocephaly | Gross Motor | 156 | -3.21 (-8.39 to 1.97) | 0.223 | 0.498 |
|  | Fine Motor | 156 | -5.68 (-10.74 to -0.62) | 0.028 | 0.141 |
|  | Adaptive Behavior | 156 | -7.16 (-12.64 to -1.68) | 0.011 | 0.119 |
|  | Language | 156 | -3.36 (-7.58 to 0.86) | 0.118 | 0.394 |
|  | Personal-Social | 156 | -2.66 (-6.96 to 1.64) | 0.224 | 0.498 |
| Scaphocephaly | Gross Motor | 156 | -5.26 (-14.46 to 3.94) | 0.260 | 0.502 |
|  | Fine Motor | 156 | -4.94 (-14.35 to 4.47) | 0.301 | 0.502 |
|  | Adaptive Behavior | 156 | 0.21 (-9.22 to 9.65) | 0.964 | 0.964 |
|  | Language | 156 | 2.71 (-4.90 to 10.31) | 0.483 | 0.743 |
|  | Personal-Social | 156 | -0.72 (-7.67 to 6.23) | 0.837 | 0.881 |
| Asymmetric brachycephaly | Gross Motor | 156 | -2.79 (-11.69 to 6.10) | 0.536 | 0.766 |
|  | Fine Motor | 156 | -1.07 (-7.87 to 5.72) | 0.755 | 0.881 |
|  | Adaptive Behavior | 156 | 0.69 (-5.37 to 6.76) | 0.821 | 0.881 |
|  | Language | 156 | -1.01 (-7.48 to 5.46) | 0.758 | 0.881 |
|  | Personal-Social | 156 | -1.18 (-6.21 to 3.86) | 0.645 | 0.86 |

*Reference category was infants with normal cranial morphology. Models were adjusted for infant sex, gestational age, age at Gesell assessment, maternal education level, and pregnancy complications where applicable.P FDR values were obtained by applying the Benjamini–Hochberg false discovery rate correction globally across all 20 tests within each sensitivity scenario.CI confidence interval, FDR false discovery rate, GA gestational age at birth. BWZ, birth-weight-for-gestational-age z-score.*P FDR < 0.05.*
